# Occupational Health and Safety and Turnover Intention in the Ghanaian Power Industry: The Mediating Effect of Organizational Commitment

**DOI:** 10.1155/2019/3273045

**Published:** 2019-09-24

**Authors:** Suxia Liu, Emmanuel Gyabeng, Gilbert Joshua Atteh Sewu, Nana Kwame Nkrumah, Bright Dartey

**Affiliations:** School of Management, Jiangsu University, Zhenjiang, China

## Abstract

This study aimed at investigating the effect of occupational health and safety (OHS) on employee's turnover intention (TI) with the mediating effect of organizational commitment (OC) in the Ghanaian power industry. *Methods*. With stratified sampling technique, 350 participants were selected to participate in the study with standardized quantitative questionnaires to measure the variables involved in the study and SmartPLS 3-structural equation modeling technique to analyze the data. *Results*. The results showed that (1) occupational health and safety and turnover intention are negatively related (*β* = 0.245, *p* < 0.05); (2) there exists a positive relationship between occupational health and safety and organizational commitment (*β* = 0.820, *p* < 0.05); (3) organizational commitment and turnover intention are negatively related (*β* = 0.640, *p* < 0.05); and (4) organizational commitment significantly mediates the relationship between occupational health and safety and turnover intention (indirect effect = −0.53 and direct effect = −0.25, *p* < 0.05). *Conclusion*. Employees satisfied with the health and safety system of their organization tend to be committed to their organization and have low turnover intention, and vice versa.

## 1. Introduction

The significance of the power industry cannot be downplayed in any country as it is the pivot of all infrastructural and socioeconomic development. Power is critical for economic growth and serves as the lifeline of the economy of every country [1]. All other sectors of the economy like the banking, health, communication, transportation, and mining are equally important; nonetheless, their survival and operations are heavily dependent on power. The power industry has been defined as a professional industry responsible for the generation, transmission, and distribution of power or electricity. The Volta River Authority (VRA) mentioned in this study is a state-owned power engineering company that generates and sells about 85% of power capacity to Ghana and other parts of Africa such as Togo and Benin. Ghana has high electricity access rate of over 83 percent (2016), second only to South Africa in sub-Saharan Africa [1]. VRA currently manages the hydropower assets and part of the thermal generation capacity while Independent Power Producers (IPPs) account for only 15 percent of installed generation capacity. It is estimated that the major power crisis in 2006–2014 has reduced the country's GDP growth by one percent on a yearly basis [2], which indicates the pivotal role the power generation sector plays in feeding the growth of the Ghanaian economy. Despite the major and significant role, the power industry plays in the growth of the economy, and it also presents major challenges specifically with respect to occupational health and safety for employees in the industry. In comparison with other sectors, the operations of the power sector are quite complex and unique. The nature of the operations and the type of chemical products produced pose chemical, biological, and mechanical risk factors and health-related issues that can have a serious impact on the physical well-being of employees working in such an environment [3]. It is therefore very important for management of this sector to be more complacent and critical in providing a safe and healthy work environment that will reduce risk exposure surrounding their employees. Occupational health and safety has in recent times gained worldwide attention as it has been linked to job satisfaction, productivity, organizational commitment, and turnover intention of employees [4, 5]. Turnover intention issue among employees is one of the key concerns of top management as the hiring and training of new staff require more resources, time, money, knowledge, and so on [6]. One major challenge modern organizations face is turnover [7]. In this regard, keeping good and experienced staff should be a topmost priority in a much significant sector such as the power industry.

Some researchers have identified that organizational commitment is one of the key factors influencing turnover intention among employees. Amponsah-Tawiah et al. [8] found that there exists a positive and significant relationship between occupational health and safety management and how workers respond affectionately and normatively which translates into higher level of commitment towards their job. Retaining employees in the Ghanaian power industry has become a herculean task to deal with by management as a seemingly huge percentage of the workforce find their way out of the industry. According to Leblebici [9], safety and health factors affect employees' desire to remain with an organization. Turnover in the Ghanaian power industry is on the increase. Could this increased rate of turnover be associated with the perceived inadequate health and safety structures in the power industry? In defiance of the stunning contribution of the power sector and the health and safety risks its employees are exposed to, coupled with the increasing turnover rate, arguably no or little empirical research has been focused on this sector in Ghana and sub-Saharan Africa at large as the few researches on occupational health and safety tend to focus on other sectors such as health, transport, and mining. This study aims at overcoming this gap by investigating the variables involved and also establishing the relationship among these variables. The purpose of this research therefore is to examine the relationship among occupational health and safety, organizational commitment, and turnover intention and test the effect of organizational commitment as the mediating variable on occupational health and safety and turnover intention.

## 2. Literature Review and Hypothesis Development

### 2.1. Occupational Health and Safety

OHS is a cross-disciplinary area concerned with protecting the safety, health, and welfare of people engaged in work or employment. A joint definition of occupational health by ILO and WHO [10] stated that “occupational health should aim at: the promotion and maintenance of the highest degree of physical, mental, and social well-being of workers in all occupations; the prevention amongst workers of departures from health caused by their working conditions; the protection of workers in their employment from risks resulting from factors adverse to health; the placing and maintenance of the workers in an occupational environment adapted to their physiological and psychological capabilities; and, to summarize: the adaptation of work to man and of each man to his job.” Occupational health refers to the identification and control of the risks arising from physical, chemical, and other workplace hazards in order to establish and maintain a safe and healthy working environment. However, occupational safety focuses on the protection of workers from physical injuries [11]. Mejia et al. [12] assert that occupational health and safety is a broad area which includes both physical and emotional well-being. Effectively managing workplace safety and health requires far more than reducing the number of job-related accidents and injuries. Health and safety is a positive concept that includes social and personal resources as well as physical capabilities [13].

Jackson et al. [14] explain the term occupational health and safety as the physiological-physical and psychological conditions of a workplace that result from work environment provided by the organization. Physiological-physical conditions include diseases and accidents such as the actual loss of life, repetitive motion injuries, back pain, and carpal tunnel syndrome. Psychological conditions encompass symptoms of poor mental health and job burnout, including apathy, emotional exhaustion, withdrawal, confusion about roles and duties, mistrust of others, inattentiveness, irritability, and a tendency to become distraught over trifles. These conditions often are responses to workplace stress and low quality of work life. According to the WSHI [15], more than 2.78 million people die each year globally through workplace injuries and accidents. Additionally, there are some 374 million nonfatal work-related injuries and illnesses each year. Many of these result in extended absences from work as a result of unsafe or unhealthy workplace conditions every year. Work-related health problems have increased since the 1990s, and they could indirectly inhibit productivity [16]. The Labour Department of Ghana [17] gave a total of 8,692 work-related accidents reported to the Department for compensation claims, while the 1999 figure stood at 4,088. This figure represents only those occurring in the formal sector. Kaynak et al. [18], by examining the effect of occupational health and safety on organizational commitment, work alienation, and job performance in their study, found that safety procedure and safety support have a significant positive relationship with organizational commitment. Amponsah-Tawiah and Mensah [19] found that there exists a positive and significant relationship between occupational health and safety management and how workers respond affectionately and normatively which translates into a higher level of commitment towards their job. People are willing to go a long way to reciprocate to a caring organization to the point of committing themselves to the values of the organization [20, 21]. Calisir et al. [22] found that job satisfaction, organizational commitment, job stress, and role ambiguity are significant determinants of turnover intention. Management commitment to safety has a positive relationship with organizational commitment, job satisfaction, and organizational performance. However, there is a negative relationship between management commitment to safety and turnover intention [23]. Firth et al. [24] also established that the relationship between managers and employees also influences turnover intention. Perceived supervisor safety support has a significant effect on turnover [25]. Aytac et al. [26] also found turnover intention among employees who had experienced workplace violence. According to VRA [27], the total reported occupational accidents were thirteen (13) which was higher than that reported in 2015 which was four (4) representing 69% increase over 2015.

Based on the literature review, this study hypothesizes the following:

  H1: there is a significant negative relationship between occupational health and safety and turnover intention
  H2: there is a significant positive relationship between occupational health and safety and organizational commitment

### 2.2. Organizational Commitment

Organizational commitment is of much significance to scientific researchers and managers of organizations and institutions [28]. According to Ehrhardt et al. [29], organizational commitment is “a state in which an employee identifies with a particular organization and its goals and wishes to maintain membership in the organization.” Organizational commitment is therefore the degree in which an employee is willing to maintain membership due to interest and association with the organization's goals and values. Commitment is a force that binds an individual to a course of action of relevance to one or more targets [30]. Organizational commitment has been related to valuable outcomes for both employees and employers that could result in enhanced feelings of belonging, security, efficacy, greater career advancement, increased compensation, and increased intrinsic rewards for the individual [31]. In their study of occupational health and safety and organizational commitment in the Ghanaian mining industry, Amponsah-Tawiah and Mensah [19] found that there exists a positive and significant relationship between occupational health and safety management and how workers respond affectionately and normatively with a very higher level of commitment towards their job. They further explained that workers who feel exposed to greater risk may resign from their respective jobs which feed into reduction in the labor workforce. Organizational commitment contributes uniquely to turnover intention [32] Yussof et al. [33] found that employees' affective and normative commitment has a negative relationship with turnover intention. Chen [34] also confirmed that organizational commitment has a negative impact on employees' intention. Highly committed employees wish to stay with their employing organizations [35]. Wong and Spence Laschinger [36] also found a negative relationship between organizational commitment and turnover intention. There exists a negative relationship between organizational commitment and turnover intention [37]. Similarly, Ahmed et al. [38] also found a negative relationship between organizational commitment and turnover intention. In their study of factors affecting turnover intention among academicians in the Malaysian Higher Education Institution, Saraih et al. [39] found a negative correlation between organizational commitment and employee turnover intention. Turnover intention is highly influenced by affective commitment [40]. Based on the literature review, this study hypothesizes the following:

  H3: there is a significant negative relationship between organizational commitment and turnover intention
  H4: organizational commitment significantly mediates occupational health and safety and turnover intention

### 2.3. Turnover Intention

**Figure 1:**
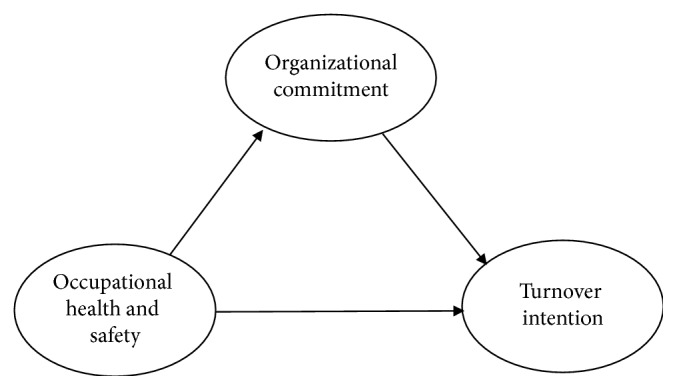
Research model.

Turnover intention refers to employee's desire or attempt to leave the organization [41]. Yussof et al. [33] defined turnover intention as a voluntary decision to leave the organization. Employee's turnover intention consists of employees voluntarily or involuntarily leaving their company [42]. Turnover intention is employees' ways to withdraw from a company because she can no longer identify with the work [43]. According to Saridakis and Cooper [44], there are four main steps involved in the process of turnover intention as the employee (1) assesses the current job, (2) evaluates his or her level of satisfaction with the company and the job, (3) evaluates the costs and consequences associated with leaving the company, and (4) assesses the alternative jobs available to compare and contrast the pros and cons of each. According to Shipp et al. [45], higher turnover intention may imply that employees are dissatisfied, unengaged, distracted, or unproductive. The best way to predict actual turnover is turnover intention [30]. Calisir et al. [22] found that job satisfaction, organizational commitment, job stress, and role ambiguity are significant determinants of turnover intention. Aytac et al. [26] also found high turnover intention among employees who had experienced workplace violence. Firth et al. [24] also established that the relationship between managers and employees also influences turnover intention. Brown et al. [46] found that organizational factors such as supervision, management, and company culture as well as personal factors such as career goals and family circumstances also influence turnover intention. The model of the study is presented in Figure 1.

## 3. Materials and Methods

The quantitative research approach was employed for this study. The research model for this study was validated by using partial least squares (SmartPLS 3) structural equation modeling technique. The measurement items for this study were adopted from previous works. The measurement items for occupational health and safety were adopted from Hayes et al. [47] Workplace Safety Scale (WSS) which measures five specific constructs, each with 10 items. These constructs include job safety (sample item “my job is dangerous”), coworkers' safety (sample item “my coworkers look out for others safety”), supervisor safety (sample item “my supervisor trains workers to be safe”), management safety (sample item “management investigates safety problems quickly”), and safety programs (sample item “the safety programs and policies are sufficient to prevent accidents”). Organizational commitment measuring items was adopted from Allen and Meyer [48] Organizational Commitment Questionnaire (OCQ). The OCQ has three distinct constructs, namely, affective commitment (sample item “I would be happy to spend the rest of my career with this organization”), continuance commitment (sample item “right now, staying with my organization is a matter of necessity as much as desire”), and normative commitment (“I owe a great deal to my organization”). Turnover intention measures were adopted from Bothma and Roodt [43] Turnover Intention Scale-6 (sample item “I have always considered leaving my job”). However, the scales were modified to suit the context of this study. All items were measured by using a 5-point Likert scale ranging from “strongly disagree” to “strongly agree.”

The study was carried out through the survey method with questionnaire as the main instrument. The data were collected from the employees of the Volta River Authority with more attention given to the engineers and technicians because their job is considered relatively more dangerous in comparison with other departments. In compliance with research ethics, permission was sought from the authorities of this organization to collect data from the employees. With a total population of 3500, Krejcie and Morgan [49] formula table for determining sample size from a given population was used to obtain a sample size of 350 for this study. However, 400 questionnaires were distributed in order to get the exact sample size needed as proposed by Krejcie and Morgan [49] and the 350 needed questionnaires were successfully retrieved in one month. The study used the stratified sampling technique because, in comparison with other employees, relatively more engineers and technicians were required for the study. The data collection was done within one month.

## 4. Results and Findings

Table 1 provides the distribution of respondents' demographic characteristics. Out of the 350 participants, 133 (38%) were between the ages of 31 and 40, which was the highest of the age groups ranging between “below 21 years” and “60 years.” 215 (61.4%) were males and 135 (38.6%) were females. With respect to the level of education, 148 (42.3%) were first degree holders, which was the highest. 123 (35.1%) had worked with the company for 6 to 10 years. 277 (79.1%) were full-time workers and 73 (20.9%) were part-time workers. 134 (38.3%) were engineers and 102 (29.1%) were technicians. The others constituted the rest of the percentage.

### 4.1. Measurement Model

In analyzing the strength of the measurement model, partial least squares (SmartPLS 3) was used because of the complex nature of the model and for the fact that PLS neither produces inadmissible solutions nor suffers factor indeterminacy [50]. It is also good for meditational analysis [51] as well as its predictive strength [52]. PLS does not provide an established global goodness-of-fit criterion [53]. However, a systematic two-step process encompassing the assessment of the measurement model and the structural model can be used to measure the quality of the model [54, 55]. Based on the above recommendation, convergent validity and discriminant validity were used to validate the measurement model. However, composite reliability was used to test the internal consistency reliability since it covers some shortcomings of the Cronbach alpha (CA) [53, 56]. Again, composite reliability takes into account that indicators have different loadings [57]. Nevertheless, all the variables met the threshold for the Cronbach alpha (0.7) ranging from 0.882 to 0.965.

Fornell and Bookstein [50] proposed method of testing convergent reliability was adopted; (1) all the indicators should be significant (minimum of 0.05) and their factor loadings should be greater than 0.7; (2) the average variance extracted (AVE) value should be greater than 0.05. The table shows all the indicators are highly significant with their loadings all above the required threshold ranging from 0.863 to 0.942 with the exception of T12 with the loading of 0.573. However, as stated by Loureiro and Kastenholz [51], the construct still accounts for more than 50% of the variance in the said observed variable. Therefore, the item was not rejected. The average variance extracted value for all the constructs also exceeded the threshold, ranging from 0.602 to 0.897. Therefore, the measurement model is valid.

However, with respect to the discriminant validity, according to Fornell and Bookstein [50], the AVE of the construct should be greater than the construct's highest squared correlation with any other construct. All the values of the AVE square root are greater than the intercorrelation values between constructs. Therefore, this is also valid. Tables 2 and 3 display the loadings, internal consistency reliability (composite reliability), and average variance extracted (AVE) and the correlation matrix of the constructs, respectively.

### 4.2. Structural Model

**Figure 2:**
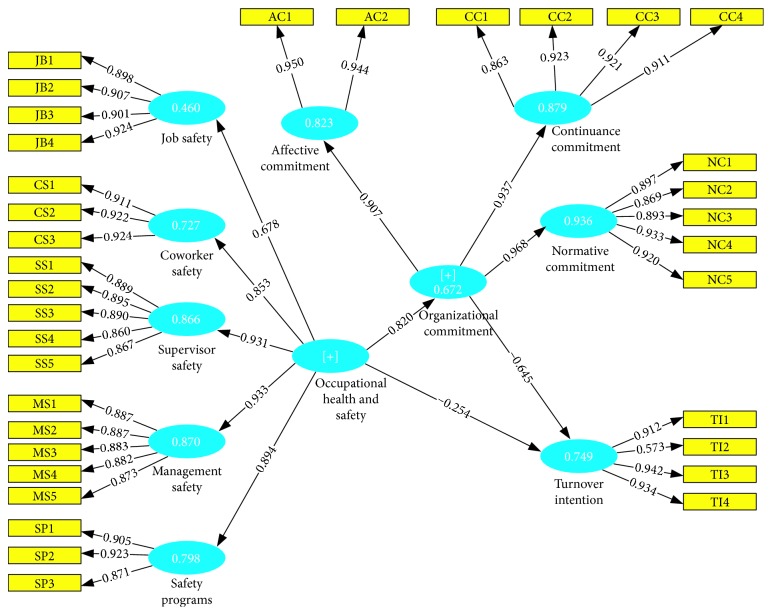
Structural equation model.

Structural equation modeling (SmartPLS 3) was used to evaluate the relationships simultaneously. From the analyses, occupational health and safety has a significant positive influence on organizational commitment (*β* = 0.820, *t* = 40.830, *p* < 0.05). Again, occupational health and safety has a significant negative relationship on turnover intention (*β* = 0.254, *t* = 3.980, *p* < 0.05), and finally, organizational commitment has a significant negative relationship on turnover intention (*β* = 0.64, *t* = 9.66, *p* < 0.050). In total, the model explains 74.9% of the variance in turnover intention and occupational health and safety explains 67.2% of the variance in organizational commitment. The structural equation model is presented in Figure 2.

### 4.3. Mediating Analysis

An analysis was conducted to test the mediating effect of organizational commitment on occupational health and safety and turnover. The bootstrapping procedure was used to test the effect of the intervening variable. Bootstrapping was adopted ahead of the popular causal step approach of which Baron and Kenny [51] procedure plays a major role because the causal step approach has been criticized by many recent researchers for the fact that it is not based on a quantification of the very thing it is attempting to test which is the mediating effect [58, 59].

Bootstrapping is the most preferred and valid method for testing intervening variable's effect since it generates an empirical representation of the sampling distribution of the indirect effect by treating the obtained sample of size “*n*” as a representation of the population [59, 60]. From the analysis, the introduction of organizational commitment reduced the coefficient value of path OHS to TI from −0.78 to −0.254. The results of the mediating analysis show a significant indirect effect between the predictor and the outcome. Partial mediation occurs when the insertion of the mediating variable significantly reduces the strength of the relationship (coefficient) between the independent variable and the dependent variable [51]. Therefore, a significant mediating relationship is proven. The result of the mediating analysis is shown in Table 4.

## 5. Discussion and Conclusion

Based on the findings of the research, turnover intention is directly influenced by occupational health and safety. Organizational commitment significantly mediates the relationship between occupational health and safety and turnover intention. The findings of the research prove that occupational health and safety has a direct significant negative relationship with turnover intention (*β* = −0.245, *t* = 3.98, *p* < 0.05). This result corroborates the findings of Michael et al. [23] whose study established a negative relationship between occupational health and safety and turnover intention. It is also consistent with previous studies by [8, 24, 26]. This can simply be explained that employees who perceive their work is safe or work in a safe and healthy environment will be willing to remain with their organization while it is more likely for employees who feel the occupational health and safety system in their organization is not worthwhile to quit the organization. This finding supports hypothesis 1 that there is a significant negative relationship between occupational health and safety and turnover intention. Moreover, from the analysis, occupational health and safety is seen to have a direct positive influence on organizational commitment (*β* = 0.820, *t* = 40.83, *p* < 0.05). This finding is consistent with Amponsah-Tawiah and Mensah [19] whose research showed that there exists a positive relationship between occupational health and safety and how workers respond affectionately and normatively with a very high level of commitment towards their job. It is also consistent with other previous researches [18, 23]. The findings support hypothesis 2 that there exists a significant positive relationship between occupational health and safety and organizational commitment. This implies that employees will be committed to an organization where workplace safety and health is prioritized and vice versa. Furthermore, from the analysis, organizational commitment is shown to have a significant negative influence on turnover intention (*β* = −0.64, *t* = 9.66, *p* < 0.05). This corroborates the findings of previous studies [33, 34, 36, 38, 39]. This implies that employees who are committed to their job find it difficult leaving their organization and vice versa. The result of the mediating analysis shows that organizational commitment significantly mediates the relationship between occupational health and safety and turnover intention with a total indirect effect of −0.53. Thus, the insertion of the mediating variable reduced the path coefficient of OHS and TI from −0.78 to −0.25. This may imply that employees who feel satisfied with the health and safety conditions in their organizations tend to be committed to the organization and, thereby, have low turnover intention and vice versa. Therefore, this finding attests that organizational commitment significantly mediates the relationship between occupational health and safety and turnover intention. This also supports hypothesis 4.

The overall findings of the study imply that employees who feel satisfied with the health and safety system of their organization tend to be committed to their organization and intend to be with the organization for a long time. Conversely, employees who are not satisfied with the health and safety system of their organization tend to be uncommitted and consequently quit the organization. Considering this, the study recommends that the management of organizations should show utmost concern for health and safety issues and ensure that the necessary and required policies and programs are implemented and enforced. The positive perception of employees on the workplace safety will definitely propel employees to be committed as well as reduce their turnover intention.

Even though previous studies have in one way or the other established relationships between occupational health and safety and organizational commitment, occupational health and safety and turnover intention, as well as organizational commitment and turnover intention, none are focused on testing the mediating effect of organizational commitment on occupational health and safety and turnover intention. This study therefore fills this gap in the literature and provides empirical proof of the effect of the mediating variable on the predictor and the outcome. Understanding the relationships and the role organizational commitment playing in occupational health and safety and turnover intention may help the management of organizations in making decisions and policies that improve the workplace safety system and, accordingly, positively influence employees' commitment to the organization and consequently reduce their turnover intention.

The study made use of self-report data which are likely to suffer biases such as acquiescence and social desirability from respondents and can in turn influence the findings of the study. Besides, the Volta River Authority has quite a number of subsidiaries across the country. However, this study only focused on the parent entity (holding company) which is likely to have improved standards and better workplace safety and health conditions in comparison with the subsidiaries. Therefore, future studies can focus on the subsidiaries using the same variables.

## Figures and Tables

**Table 1:** Respondents' demographic characteristics.

Variable	Characteristics	Frequency	Percent
Age (y)	Below 21	3	0.9
	21–30	62	17.7
	31–40	133	38
	41–50	113	32.3
	51–60	39	11.1

Gender	Male	215	61.4
	Female	135	38.6

Level of education	SSCE/WASSCE/technical certificate	31	8.9
	Higher national diploma	63	18
	First degree	148	42.3
	Master's degree and above	108	30.9

Number of years participants have worked for the company (y)	Below 1	9	2.6
	1–5 yrs	79	22.6
	6–10 yrs	123	35.1
	11–15 yrs	97	27.7
	20 yrs and above	42	12

Job title	Laborer	38	10.9
	Technician	102	29.1
	Engineer	134	38.3
	Others	76	21.7

Terms of employment	Full-time	277	79.1
	Part-time	73	20.9

**Table 2:** Factor loadings, means, standard deviations, reliabilities, and average variance extracted.

Construct		Item	Mean	SD	Factor loading
Job safety (JB) CR = 0.949 AVE = 0.823	JB1	My job is dangerous	3.050	0.969	0.898
	JB2	I sometimes think my job is unhealthy	2.920	0.922	0.907
	JB3	I fear for my health	2.960	0.961	0.901
	JB4	My job poses a chance of death	2.930	0.912	0.924

Coworker safety (CS) CR = 0.942 AVE = 0.845	CS1	My coworkers follow safety rules	3.580	0.938	0.911
	CS2	My coworkers keep work area clean	3.680	0.922	0.922
	CS3	My coworkers look out for others safety	3.640	0.953	0.924

Supervisor safety (SS) CR = 0.945 AVE = 0.775	SS1	My supervisor trains workers to be safe	3.620	0.915	0.889
	SS2	My supervisor enforces safety rules	3.570	0.957	0.895
	SS3	My supervisor praises safe work behaviors	3.510	0.978	0.89
	SS4	My supervisor rewards safe work behaviors	3.470	1.006	0.86
	SS5	My supervisor involves workers in setting safety goals	3.550	0.974	0.867

Management safety (MS) CR = 0.946 AVE = 0.779	MS1	Management provides enough safety training programs	3.650	1.01	0.887
	MS2	Management conducts frequent safety inspections	3.600	0.993	0.887
	MS3	Management investigates safety problems quickly	3.580	0.977	0.883
	MS4	Management provides all needed safety equipment	3.520	0.983	0.882
	MS5	Management rewards safe workers	3.450	0.985	0.873

Safety programs (SP) CR = 0.927 AVE = 0.81	SP1	The safety programs and policies in this organization are worthwhile	3.650	0.924	0.905
	SP2	The safety programs and policies in this organization are useful and effective	3.650	0.901	0.923
	SP3	The safety programs and policies are sufficient to prevent accidents	3.520	0.969	0.871

Affective commitment (AC) CR = 0.946 AVE = 0.897	AC1	I would be happy to spend the rest of my career with this organization	3.390	1.104	0.95
	AC2	I really feel as if this organization's problems are my own	3.500	1.088	0.944

Continuance commitment (CC) CR = 0.948 AVE = 0.819	CC1	Right now, staying with my organization is a matter of necessity as much as desire	3.300	1.102	0.863
	CC2	It would be very hard for me to leave my organization right now, even if I wanted to	3.320	1.092	0.928
	CC3	Too much of my life would be disrupted if I decided I wanted to leave my organization now	3.230	1.108	0.921
	CC4	I feel that I have too few options to consider leaving this organization	3.380	1.066	0.911

Normative commitment (NC) CR = 0.957 AVE = 0.815	NC1	Even if it were to my advantage, I do not feel it would be right to leave my organization now	3.270	1.079	0.897
	NC2	I would feel guilty if I left my organization now	3.170	1.063	0.869
	NC3	This organization deserves my loyalty	3.570	1.068	0.894
	NC4	I would not leave my organization right now because I have a sense of obligation to the people in it	3.490	1.088	0.933
	NC5	I owe a great deal to my organization	3.560	1.082	0.92

Turnover intention (TI) CR = 0.913 AVE = 0.73	TI1	I have always considered leaving my job	2.500	1.248	0.912
	TI2	I am frustrated when not given the opportunity to achieve personal work-related goals	2.850	0.911	0.573
	TI3	I often dream about getting another job that will better suit my personal interest	2.690	1.222	0.942
	TI4	It is likely for me to accept another job at the same compensation level should it be offered to me	2.640	1.144	0.934

**Table 3:** Correlation matrix among constructs.

	AC	CC	CS	JB	MS	NC	OHS	OC	SP	SS	TI
AC	**0.947**										
CC	0.787	**0.937**									
CS	0.659	0.585	**0.919**								
JB	0.527	0.493	0.495	**0.907**							
MS	0.755	0.674	0.754	0.503	**0.933**						
NC	0.849	0.841	0.656	0.549	0.742	**0.968**					
OHS	0.809	0.72	0.853	0.678	0.833	0.802	**0.931**				
OC	0.907	0.905	0.669	0.555	0.763	0.903	0.820	**0.862**			
SP	0.734	0.655	0.716	0.499	0.841	0.717	0.894	0.740	**0.900**		
SS	0.776	0.676	0.751	0.541	0.838	0.764	0.823	0.779	0.794	**0.880**	
TI	0.801	0.794	0.627	0.521	0.742	0.817	0.783	0.853	0.703	0.741	**0.854**

*Note.* Square root of AVEs is given in diagonal in bold. AC = affective commitment; CC = continuance commitment; CS = coworker safety; JB = job safety; MS = management safety; NC = normative commitment; OHS = occupational health and safety; OC = organizational commitment; SP = safety policies; SS = supervisor safety; TI = turnover intention.

**Table 4:** Results of the mediating analysis.

Effect	Path	Original sample (O)	Sample mean	SD	T stats	*p* value
Total effects	OHS–OC	0.82	0.82	0.02	40.83	0.0001
	OC–TI	−0.65	−0.65	0.07	9.66	0.0001

Total indirect effect	OHS–TI	−0.78	−0.78	0.02	36.38	0.0001
	OHS–TI	−0.53	−0.54	0.06	9.08	0.0001

Specific indirect effect	OHS–OC–TI	−0.53	−0.54	0.06	9.08	0.0001

*Note*. OHS = occupational health and safety; OC = organizational commitment; TI = turnover intention.

## Data Availability

The data used to support the findings of this study are available from the corresponding author upon request.
